# Immediate orthodontic load on dental implants: an option for adult treatment

**DOI:** 10.1590/2177-6709.24.6.069-079.bbo

**Published:** 2019

**Authors:** José Augusto Mendes Miguel, Tatiana Ettore do Valle de Sousa Freitas

**Affiliations:** 1 Universidade do Estado do Rio de Janeiro (Rio de Janeiro/RJ, Brazil).; 2 Universidade Federal do Rio de Janeiro (Rio de Janeiro/RJ, Brazil).; 3 Odontoclínica Central da Marinha do Brasil (Rio de Janeiro/RJ, Brazil).

**Keywords:** Orthodontic anchorage, Dental implants, Immediate load

## Abstract

The demand for orthodontic treatment in adults has been increasing. However, these patients often require a multidisciplinary approach, due to the lack of posterior teeth, requiring additional anchorage. The skeletal anchorage by endosseous implants is an option, since they may be used later for prosthetic rehabilitation. The application of immediate load on these appliances for orthodontic movement may reduce the costs and total treatment time. This paper discusses the utilization of endosseous dental implants with immediate load for absolute anchorage for orthodontic movement, with later utilization for prosthetic rehabilitation.

## RESUMO

A demanda por tratamento ortodôntico em pacientes adultos tem sido cada vez maior. Entretanto, esses pacientes frequentemente exigem um tratamento multidisciplinar, devido às ausências de dentes posteriores, necessitando de ancoragem adicional. A ancoragem esquelética usando implantes osseointegrados apresenta-se como alternativa, uma vez que eles serão posteriormente utilizados para reabilitação protética. A aplicação de carga imediata sobre esses dispositivos, para movimentação ortodôntica, pode reduzir custos e o tempo total de tratamento. O presente artigo tem como objetivo discutir o emprego de implante dentário osseointegrado, com carga imediata, como ancoragem absoluta para movimentação ortodôntica e, posteriormente, utilizado para reabilitação protética.

## INTRODUCTION

The demand for orthodontic treatment in adults has been increasing, especially due to the increased esthetic demands^1^ ^,^ ^2^ ^,^ ^3^ and the evolution of orthodontic appliances, which are more comfortable. However, these patients present some peculiarities, since they are often affected by periodontal disease^4^ ^,^ ^5^ and frequent tooth losses at the posterior region, which may impair the achievement of orthodontic anchorage during treatment^6^. Also, adult patients present well-defined expectations concerning the treatment, requesting a shorter treatment time able to solve their complaints^7^.

The skeletal anchorage is an option, since it does not allow movement of the anchorage unit, providing optimization of results, with utilization of simpler mechanics and reduced treatment time. For that purpose, the utilization of orthodontic mini-implants, inserted specifically during orthodontic treatment and removed thereafter, has been widely diffused.^8^ ^,^ ^9^

However, in patients presenting tooth losses that will be rehabilitated with prostheses supported by endosseous implants concomitantly to orthodontic treatment, the utilization of these implants with immediate occlusal load allows prosthetic rehabilitation early at treatment onset, while also providing the necessary anchorage to move the adjacent teeth,^10^ ^-^ ^12^ eliminating the need of temporary anchorage at the site (miniscrews or miniplates). Even though the immediate load on implants for prosthetic purposes has been widely used and investigated,^13^ the same does not apply for non-occlusal orthodontic forces (lateral). Few studies have evaluated the effects of orthodontic forces on implants considered able to receive immediate occlusal load,^6^ raising great resistance from implantologists in their indication. There is also no consensus on the healing period required for application of orthodontic force.^14^ ^-^ ^19^

Dental implants are reliable accessories for oral rehabilitation, and their utilization as anchorage units has been reported as successful in several clinical situations.^20^ ^,^ ^21^ In patients with multiple losses of posterior teeth, the utilization of endosseous implants provides better pre-prosthetic positioning of remaining teeth, enhances the orthodontic movement and reduces the undesirable side effects. They may also be used for prosthetic rehabilitation of edentulous areas, reducing the total time and treatment cost.

It should be highlighted that the utilization of endosseous implant first as anchorage and later as prosthetic abutment requires an integrated and careful multiprofessional treatment planning between Orthodontics, Surgery, Periodontology and Prosthodontics, considering the prosthetic space, need of tooth movement, shape, position and contour of the future prosthetic crown.^14^ ^,^ ^22^ Thus, it is fundamental to place it in ideal positioning to allow the desired orthodontic movement and favorable occlusal and esthetic results.

The decision on the ideal timing for load application is a clinical parameter that should be adjusted for each patient. Huang et al,^21^ who revised the literature about the concepts of anchorage involving endosseous implants, stated that direct orthodontic forces of approximately 300g generate lower stress on the implant, due to the low magnitude. They also considered that factors as surgical technique, primary stability achieved during implant insertion and quality and quantity of cortical and cancellous bone tissue of the patient should be considered.

Marins et al^23^ conducted peri-implant evaluation of endosseous implants submitted to orthodontic forces and achieved success on 100% of implants submitted to 200cN forces, indicating that they may be safely used for prosthetic rehabilitation after orthodontic finalization. Similarly, Cravero and Ibañez^11^ achieved 100% of success on 93 implants used in the maxilla and mandible. Palagi et al,^6^ comparing implants with primary stability submitted to immediate orthodontic loads of up to 200 grams and a control group with 4-month healing period with 2-year follow-up, concluded that the reduction in healing time did not affect the success of implants used as anchorage.

This paper discusses the utilization of endosseous dental implants with immediate load as absolute anchorage for orthodontic movement, presenting a case report with multiple losses of posterior teeth and need of anchorage reinforcement, in which one endosseous dental implant was inserted with application of immediate load for orthodontic and prosthetic purposes.

## CASE REPORT

Female Caucasoid patient, aged 47 years and 9 months, searched for orthodontic treatment with complaint about the smile esthetics. The anamnesis revealed that the patient presented blood hypertension controlled by continuous intake of antihypertensive drugs, without additional alterations in general health.

The facial examination revealed slightly convex profile with deficient chin and mandibular retrusion. The nasolabial angle was diagnosed as straight, and the mentolabial angle, as obtuse. 

**Figure 1:**
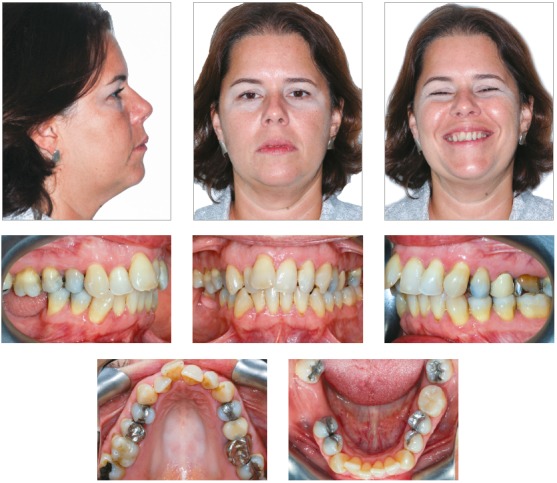
Initial facial and intraoral photographs.

The intraoral clinical examination revealed loss of teeth #37, #46 and #47, due to dental caries. The third molars were present, mesially inclined, and tooth #16 was extruded. The oral hygiene was satisfactory, yet with history of high caries incidence. The patient presented Class II division 1 left subdivision malocclusion, with overjet of 8 mm and good overbite of upper and lower incisors (2 mm). There was also severe upper (-10 mm) and lower (-5 mm) crowding at the anterior region, with upper midline deviation of 3 mm to the right side in relation to the facial midline, besides occlusal interferences in lateral and protrusive movements (Fig 1).

**Figure 2:**
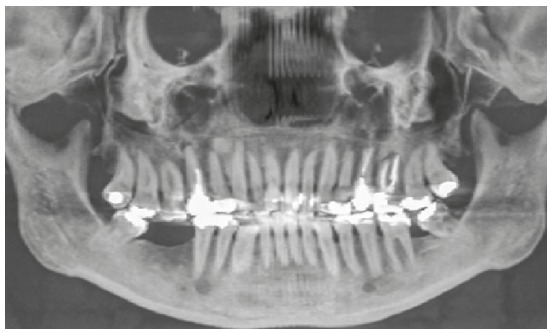
Initial panoramic radiograph.

**Figure 3:**
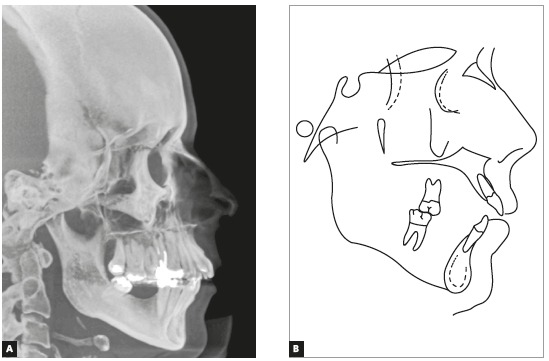
Initial lateral cephalogram (A) and cephalometric tracing (B).

The initial panoramic radiograph evidenced unsatisfactory endodontic treatment of teeth #15 and #26, with presence of periapical lesion on both teeth, besides generalized bone loss (Fig 2). The cephalometric evaluation evidenced skeletal Class II relationship (ANB = 6^o^), with well-positioned maxilla in relation to the cranial base (SNA = 82^o^) and mandibular retrognathism (SNB = 76^o^). The patient presented dolichofacial pattern (Y-axis = 63^o^ and SN.GoGn = 39^o^) and buccally tipped upper and lower incisors (1.NA = 21^o^, 1-NA = 9 mm, 1.NB = 30^o^, 1-NB = 9 mm, IMPA = 96.5^o^) (Fig 3 and Table 1).

**Table 1:** Initial and final cephalometric values.

	Measurements		Normal	A	B	A/B diff.
Skeletal pattern	SNA	(Steiner)	82°	82°	80°	2
	SNB	(Steiner)	80°	76°	76°	4
	ANB	(Steiner)	2°	6°	4°	2
	Wits	(Jacobson)	2 mm	2.5 mm	1.5 mm	1
	Angle of convexity	(Downs)	0°	10°	12°	2
	Y-axis	(Downs)	32°	39°	38°	1
	Facial angle	(Downs)	59°	63°	65°	2
	SN-GoGn	(Steiner)	87°	85.5°	83°	2.5
	FMA	(Tweed)	22°	21°	8°	13
Dental pattern	IMPA	(Tweed)	25°	30°	35°	5
	1.NA (degrees)	(Steiner)	130°	117°	131°	14
	1-NA (mm)	(Steiner)	4 mm	9 mm	2 mm	7
	$\bar{1}$.NB (degrees)	(Steiner)	4 mm	9 mm	9 mm	0
	$\bar{1}$-NB (mm)	(Steiner)	25°	29°	30°	1
	$\frac{1}{1}$- Interincisal angle	(Downs)	90°	96.5°	102°	5.5
	$\frac{1}{1}$- Apo	(Steiner)	1 mm	5 mm	4 mm	1
Profile	Upper lip - S-line	(Steiner)	0	2 mm	-1 mm	1
	Lower lip - S-line	(Steiner)	0	1 mm	2 mm	1

The treatment goals were to achieve space for alignment of the upper and lower incisors, coincide the dental midline with the facial midline, correct the overjet and achieve Class I canine relationship bilaterally, besides prosthetic rehabilitation of edentulous spaces, in interaction with specialties of Surgery, Implantology and Prosthodontics.

## TREATMENT PLANNING AND MECHANICS EMPLOYED

The multidisciplinary treatment planning comprised extraction of teeth #15 and #26, which were endodontically compromised, to achieve space for upper alignment and leveling and reduce the overjet. The plan also included placement of miniscrews as anchorage reinforcement for intrusion of tooth #16, besides insertion of endosseous implant at the region of tooth #46 to upright the tooth #48 for later prosthetic rehabilitation.

**Figure 4:**
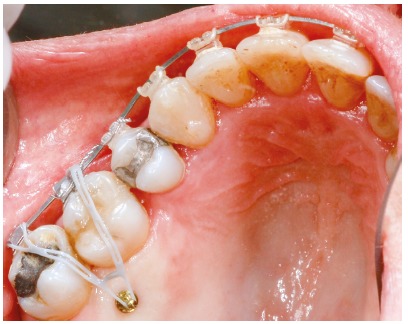
Intrusion mechanics of tooth 16 using miniscrews after space closure.

The upper arch received placement of 1.6 mm x 6 mm miniscrews (S.I.N. Implant System, São Paulo, Brazil) on the buccal and palatal aspects, between teeth #16 and #17, used as anchorage for intrusion of tooth #16, with chain elastics on the occlusal surface (Fig 4). 

**Figure 5:**
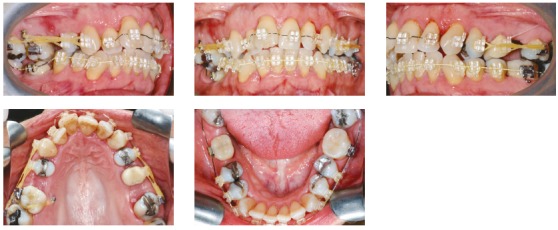
Alignment and leveling after extraction of teeth 15 and 26 and insertion of endosseous implant at the edentulous region of tooth 46 with immediate load for anchorage.

**Figure 6:**
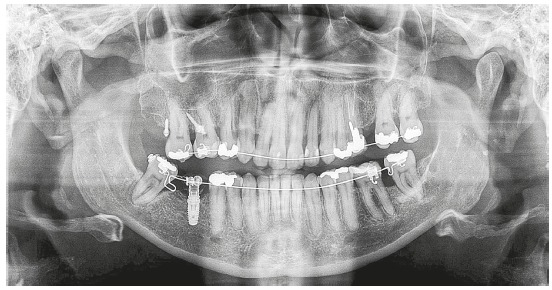
Panoramic radiograph after placement of miniscrews for intrusion of tooth 16 and endosseous implant for uprighting of tooth 48.

The lower arch received an endosseous implant at the region of tooth #46 (Figs 5 and 6), submitted to immediate load by a bracket bonded to the provisional crown, providing anchorage for uprighting and posterior mesialization of tooth #48, achieved during treatment by utilization of a power arm fabricated with 0.019 x 0.025-in stainless steel archwire. On the lower left side, the plan comprised uprighting and mesialization of tooth #38, closing the space related to absence of tooth #37. For that purpose, 0.019 x 0.025-in finalization archwires with boot-loops at the terminal end were used for uprighting of lower third molars and control of anterior and posterior torque.

## RESULTS

**Figure 7:**
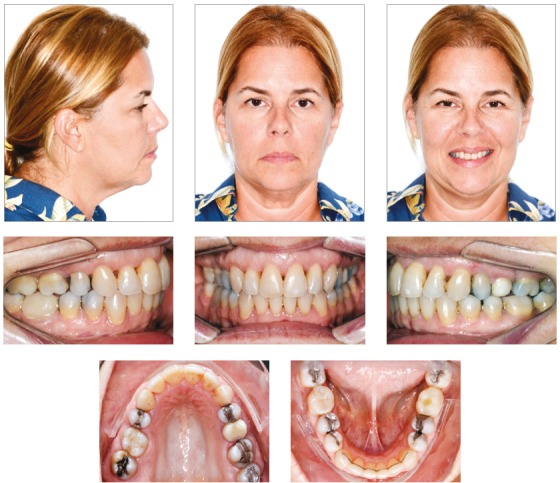
Final facial and intraoral photographs.

After finalization of orthodontic therapy, the results were evaluated at two moments: immediately after appliance removal and on the 3-year follow-up. The results achieved at treatment completion can be observed on Figure 7. The proposed objectives were achieved, evidencing enhanced smile esthetics at treatment completion, with consequent increase in the patient’s self-esteem, as well as increased masticatory efficiency, provided by correction of malocclusion.

**Figure 8:**
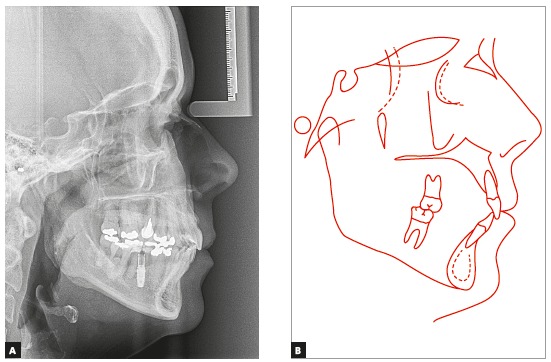
Final lateral cephalogram (A) and cephalometric tracing (B).

**Figure 9:**
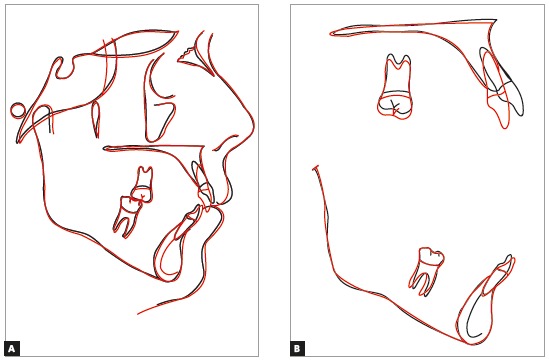
Total (A) and partial (B) superimpositions of initial (black) and final (red) cephalometric tracings.

The cephalometric analysis (Fig 8) evidenced maintenance of the vertical skeletal pattern, while in anteroposterior direction there was slight reduction of the ANB angle (from 6^o^ to 4^o^), with Class II dental compensation by retroclination of upper incisors and projection of lower incisors. These effects were expected, since the exclusively orthodontic treatment was indicated. The cephalometric tracings superimpositions revealed maintenance of palatal and mandibular planes, with exclusively dental movement, represented by projection of lower incisors and retroclination of upper anterior teeth (Fig 9).

**Figure 10:**
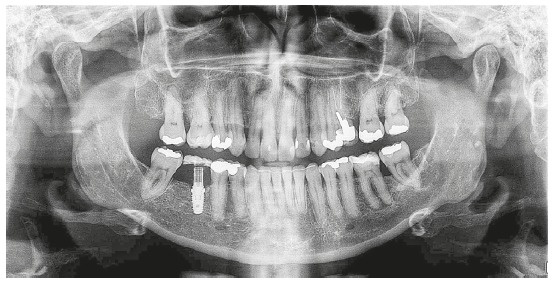
Final panoramic radiograph.

The final panoramic radiograph (Fig 10) evidences the adequate root parallelism, with correct inclination of teeth #38 and #48, allowing satisfactory prosthetic rehabilitation at the region of tooth #46, supported by the endosseous implant. The implant did not exhibit any sign of mobility or alteration in adjacent periodontal tissues at any treatment stage. Correct alignment and leveling were achieved, with correction of upper midline and overjet, and functional occlusion with incisal guidance during protrusive mandibular movement and canine disocclusion (working side), without interferences in balance on laterality movements.

**Figure 11:**
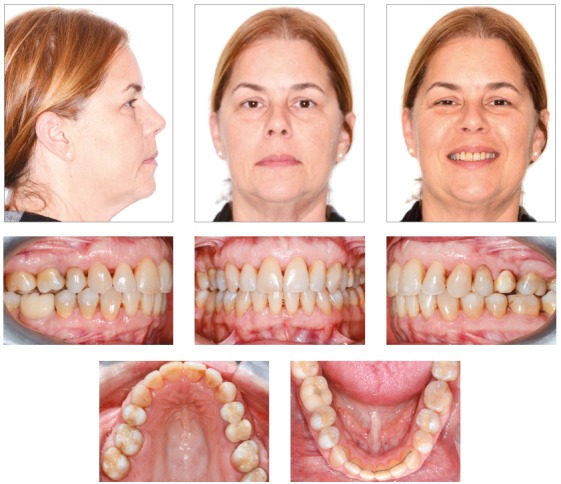
Facial and intraoral photographs 3 years after orthodontic treatment.

The results remained stable after 3-year follow-up, and can be seen in Figure 11.

## DISCUSSION

The biggest challenge in the treatment of patients with loss of multiple posterior teeth is the difficulty to achieve effective orthodontic anchorage during treatment^6^, due to the lack of anchorage teeth. In addition to temporary skeletal anchorage such as miniplates and miniscrews, endosseous implants can be used as coadjutants to orthodontic mechanics. Little has been discussed about the effects of orthodontic forces applied immediately after implant placement^6^.

In the treatment of this patient presenting loss of multiple posterior teeth in the lower arch, tooth malpositioning and lack of posterior anchorage, the therapeutic option of choice was uprighting and subsequent mesialization of tooth #48, using an endosseous implant at the region of tooth #46 as anchorage, which was submitted to immediate load by a bracket bonded to the provisional crown.

The option to mesially move the tooth #48, closing the edentulous space, was ruled out, since some investigators highlighted the difficulty of moving teeth through edentulous areas, predisposing to loss of supporting tissue and even furcation involvement, since buccolingual dimension of the tooth is often wider than the alveolar bone area.^5^ In addition, this option increases the treatment time because it is an extensive movement, with complex and difficult orthodontic mechanics, due to the lack of posterior anchorage. Other possibility would be the extraction of tooth #48, with subsequent prosthetic rehabilitation on the lower right side, which was discarded because it was not a conservative therapy and involved extensive dentures.

Therefore, due to the clinical condition presented by the patient, the selected treatment plan comprised placement of a fixed orthodontic appliance, associated to insertion of an endosseous implant to serve as anchorage unit during mesialization and uprighting of tooth #48 to replace tooth #47. Later, tooth #46 was prosthetically rehabilitated over the previously placed implant.

In the upper arch, the difficulty involved the intrusion of tooth #16. In this context, the use of miniscrews as temporary anchorage devices provided efficiency and control in the movement, without occurrence of undesired reciprocal movement. It should be emphasized that it is important to use a bilateral force system, with insertion of miniscrews on the buccal and palatal sides, to minimize the tooth inclination and the possibility of root apex resorption.^24^

For insertion of the endosseous implant in the edentulous space to serve as anchorage unit, a multidisciplinary orthodontic, prosthetic and esthetic planning was designed, considering the prosthetic space, need for tooth movement, position and size of future prosthetic crown.^14^ ^,^ ^25^

In general, the literature indicates that immediate load can be applied to implants with primary stability whose torque during surgery reached between 35 and 60 N/cm.^26^ ^,^ ^27^ Complete osseointegration may be advisable, yet it is not essential for orthodontic anchorage, yet overload should be avoided during healing.^21^

The literature also points to the use of immediate load on these implants, since the orthodontic force, of small magnitude, would cause a very mild increase in stress at the bone-implant interface.^6^ ^,^ ^26^ Marins et al^23^ found that endosseous implants submitted to orthodontic forces over a 3-year period not only remained firm, but also sustained a healthy surrounding periodontal tissue.

Thus, it can be concluded that, if planning and primary stability obtained during surgery converge to favorable characteristics for the use of immediate load, the treatment can be performed, providing the patient with comfort, speed and lower treatment costs.

In the present case, the treatment time was extended due to the mechanical complexity of closing the extraction spaces of upper posterior teeth to reduce the increased overjet, and the difficult traction of the third molar with unfavorable inclination. However, the patient benefited from the use of endosseous implant as anchorage for uprighting and mesialization movements and its later use as prosthetic abutment, with reduction in total treatment time compared to traditional anchorage. The immediate loading did not delay or prevent osseointegration, and the tooth was successfully used in the final prosthetic rehabilitation. Scientific evidence regarding its use in Orthodontics is still scarce, and further studies are necessary to correlate the anchorage in endosseous implants and the application of immediate orthodontic load.

## CONCLUSION

The utilization of endosseous implants with immediate load may be an effective option for orthodontic treatment in adults. The skeletal anchorage provided by these devices increased the treatment efficiency, besides allowing later prosthetic rehabilitation, consequently reducing the total time. Despite the success achieved in the case, there is still little scientific evidence on this issue, thus further studies are necessary to demonstrate its use in Orthodontics. 
